# Using Biosecurity Measures to Combat Respiratory Disease in Cattle: The Norwegian Control Program for Bovine Respiratory Syncytial Virus and Bovine Coronavirus

**DOI:** 10.3389/fvets.2020.00167

**Published:** 2020-04-07

**Authors:** Maria Stokstad, Thea Blystad Klem, Mette Myrmel, Veslemøy Sunniva Oma, Ingrid Toftaker, Olav Østerås, Ane Nødtvedt

**Affiliations:** ^1^Department of Production Animal Clinical Sciences, Norwegian University of Life Sciences, Oslo, Norway; ^2^Norwegian Veterinary Institute, Oslo, Norway; ^3^Department of Food Safety and Infection Biology, Norwegian University of Life Sciences, Oslo, Norway; ^4^Section for Research and Development in Primary Production, Tine SA, Oslo, Norway

**Keywords:** bovine respiratory disease, winter dysentery, disease control, population-based, prevention, BRSV, BCoV

## Abstract

Bovine respiratory disease (BRD) cause important health problems in all cattle husbandry systems. It contributes substantially to the use of antimicrobial substances and compromises animal welfare and the sustainability of the cattle industry. The existing preventive measures of BRD focus at the individual animal or herd level and include vaccination, mass treatment with antimicrobials and improvement of the animal's environment and general health status. Despite progress in our understanding of disease mechanism and technological development, the current preventive measures are not sufficiently effective. Thus, there is a need for alternative, sustainable strategies to combat the disease. Some of the primary infectious agents in the BRD complex are viruses that are easily transmitted between herds such as bovine respiratory syncytial virus (BRSV) and bovine coronavirus (BCoV). This conceptual analysis presents arguments for combatting BRD through improved external biosecurity in the cattle herds. As an example of a population-based approach to the control of BRD, the Norwegian BRSV/BCoV control-program is presented. The program is voluntary and launched by the national cattle industry. The core principle is classification of herds based on antibody testing and subsequent prevention of virus-introduction through improved biosecurity measures. Measures include external herd biosecurity barriers and regulations in the organization of animal trade to reduce direct and indirect transmission of virus. Improved biosecurity in a large proportion of herds will lead to a considerable effect at the population level. Positive herds are believed to gain freedom by time if new introduction is avoided. Vaccination is not used as part of the program. Dissemination of information to producers and veterinarians is essential. We believe that reducing the incidence of BRD in cattle is essential and will lead to reduced antimicrobial usage while at the same time improving animal health, welfare and production. Alternative approaches to the traditional control measures are needed.

## Introduction

Bovine respiratory disease (BRD) is a worldwide health concern in cattle and is one of the most common diseases in calves and young stock in all production systems. The disease is multifactorial and develops in complex interaction between factors associated with the host, the pathogens and the environment. The existing preventive measures therefore include a wide range of strategies. Despite advances of newer and better therapeutic and preventive medications, as well as efforts to improve management and optimize the environment to prevent BRD, the morbidity and mortality rates have not declined. A recent review of evolving views on BRD control measures concludes that blanket vaccination and mass treatment provides inconstant control for BRD and highlights the need to reappraise the use of these measures (1). Our question is, however, if there are alternative strategies to antimicrobial treatment and vaccines that could be effective in reducing the impact of BRD in a sustainable cattle production.

This conceptual analysis presents arguments for combatting BRD at the population level through improved external biosecurity in cattle herds. The rationale for such a program will be given by describing the current impact of BRD, the effect of the current preventive measures and the likely effect of additional biosecurity improvements. Bovine respiratory syncytial virus (BRSV) and bovine coronavirus (BCoV) are two important causative agents in BRD. The Norwegian control program for BRSV and BCoV is presented as an example of a novel and alternative strategy to prevent and reduce BRD. Challenges of such a program and relevant differences between Norway and other areas are also discussed.

## Current Impact of BRD

BRD is a common disease in cattle worldwide, both in feedlots and non-feedlot husbandry systems (2, 3). In US feedlot cattle, BRD is the most frequently reported illness (4). In Norway, it is the most commonly diagnosed disease and the most common cause of mortality in calves (5, 6).

BRD has negative effects on the animals' life and the producers' economy. It is a major cause of morbidity, mortality and economic loss in both the beef and dairy cattle industries (7, 8). Fatalities, treatment costs, and handling of sick animals contribute to the economic losses in the acute stage of an outbreak. Considerably higher number of animals are usually found to have lung-lesions at slaughter, compared to the number of clinical BRD cases in a herd. This indicates that observed clinical cases represent only part of the problem (9). The long-term consequences are also less recognized, but reduced feed conversion, growth rate and performance might contribute considerably to the total economic losses. A long-term reduction in weight gain (7 months) was seen following a BRD outbreak among bulls in Norway (10). Calves with BRD have also been found to produce less milk when they reach first lactation (11). National studies from the UK have estimated costs associated with BRD amounting to £80 million annually for the cattle industry (12). The only scientific publication where the national-level economic effect of BRD has been estimated is from France, where an epidemiology- and productivity model was used to link BRD incidence with productivity in the different cattle industry sectors (13). The authors found that eradication of BRD in beef calves would increase the whole beef sector's productivity by 4.7–5.5%, but that the benefits from eradication would differ between enterprises. For example, young bull and veal feedlot enterprises were estimated to increase in productivity by 8.7–12.8% while the breeding farms would gain less (5.1–6.0%) (13).

Antimicrobial usage in animals may affect both public health and the environment (14). BRD and mastitis are the two main causes of antimicrobial usage in cattle worldwide, and accounts for the main quantity of antimicrobials used. Respiratory disease is the most common reason for metaphylactic antibiotic therapy in the US. 71% of feedlot cattle receive antimicrobials in feed, and 13.4% are treated with injectable antimicrobials to prevent or treat BRD (4). A wide variety of antimicrobials are used, usually broad-spectrum antibiotics including those recommended for human use only (4). In Denmark, BRD accounts for 79% of antimicrobials used in veal calves and young bulls (15). Also in Norway, BRD is the main reason for therapeutic antimicrobial usage in both dairy calves and in the beef industry (16). Reduction of BRD would significantly reduce the total use of antimicrobials in the cattle industry and by that reduce the risk of antimicrobial resistance development.

Livestock contribute to the total human-induced greenhouse gas emissions, with cattle production accounting for the majority (60%) of the livestock sector's emissions (17). Practices that improve production efficiency, such as better health management, are examples of interventions that reduce greenhouse gas emissions from livestock (17). BRD is a major production–limiting disease in both the dairy and beef industry (4, 8, 18), hence reduction of BRD is a relevant intervention to reduce the emissions from the livestock sectors. Delabouglise et al. (13) also concluded that enhancing BRD control, particularly in beef breeding farms, would substantially increase the productivity of the French cattle industry, reduce its environmental impact and satisfy consumers' demand (13).

For BRD, the severity of clinical signs, the high incidence of chronic cases, and the high mortality and morbidity estimates underscore the importance of limiting BRD to improve animal health and welfare. Freedom from disease is a fundamental aspect of animal welfare.

## Todays' Preventive Strategies–are They Satisfactory?

The multifactorial nature of BRD and the global differences in production systems of beef and dairy cattle have led to a variety of prevention strategies. Common for all strategies are attempts to either improve the animal environment, strengthen the general health and host immunity and/or minimize animal exposure to the relevant pathogens. Vaccination and preventive antimicrobial medication are the most common preventive measures, with the aim to keep a low infection pressure and/or helping the host to combat infection. All preventive measures focus on the single animal or herd as the unit of interest.

### Mass Medication With Antimicrobials

Mass medication involves administering antimicrobials to groups of animals, either as preventive/prophylactic treatment or as metaphylaxis. Murray et al. (1) concluded in a review that mass medication provides inconsistent control of BRD and poses a serious concern regarding the effect on emergence of antimicrobial resistance. A meta-analysis of randomized, controlled clinical trials concluded that antimicrobial prophylaxis and metaphylaxis demonstrated moderate, yet highly variable reductions in the relative risk of BRD morbidity (19). The most substantial reductions of relative risk were from critically important broad-spectrum antimicrobials. However, metaphylactic treatment with macrolides were found to have no effect on incidence of BRD in a controlled trial (20). In addition, a high prevalence of multidrug resistant *Mannheimia haemolytica* has been found in cattle after metaphylaxis and treatment for BRD (21). Baptiste and Kyvsgaard (19) also concluded that BRD prophylaxis/metaphylaxis represents a major driver of antimicrobial consumption for highly variable short-term gains in terms of absolute risk reduction of morbidity and mortality. The use of mass medication can hardly be seen as in accordance with the current strategy to prevent antimicrobial resistance through prudent use of antibiotics recommended by the World Health Organization, United Nations, Food and Agriculture Organization and World Organization for Animal Health (14). It is therefore necessary to promote control of BRD without the use of antimicrobial mass-medication.

### Vaccination

The use of vaccines to reduce the impact of BRD in dairy and beef cattle is common practice worldwide, although the practice lacks convincing scientific support. The development of effective vaccine programmes has been challenging (1). The short duration of immunity provided by vaccines against mucosal viral infections and the need to vaccinate immunologically immature calves in the presence of maternal antibodies have led to suboptimal effect of vaccines and challenge the cost-benefit of its use (1, 22). The effect of vaccination on herd immunity depends on the efficacy of the vaccine, but also on vaccine management such as the proportion of animals vaccinated and the timing of vaccination (23). Several authors have reviewed the vaccine efficacy of BRD vaccines, with conflicting results in calves and feedlot cattle (24, 25), and a systematic review and meta-analysis assessing the effect of commercially available BRD vaccines showed no significant difference in the risk of BRD in vaccinated calves, compared to controls (26). Despite years of research and advances in vaccine development, the use of vaccines has not provided the wanted effect against BRD.

### Management to Maintain Good Animal Health and an Optimal Environment

Improvement of the environment can favor healthy development of animals with a robust immune system. Management factors including excessive handling, commingling, and movement of animals increase the risk of BRD due to stress and immunosuppression (3, 27). An important management factor is a good routine for adequate intake of colostrum (8, 28). Annual and seasonal variation in mortality rates due to BRD have been documented, with increased rates during winter (3, 29). This has been partially explained by higher animal density during confined housing, poor ventilation and inclement weather (12). Studies from Scandinavia have found that reduction of the animal density and age span in group-pens along with 1 week of isolation of new-borns from adult cows may prevent BRD (30, 31). Nevertheless, maintenance of good health alone does not result in sufficient reduction of BRD (8), and despite education and consulting of producers on optimal management strategies, it may be difficult to achieve the desired results.

## Can Increased Herd Biosecurity Prevent BRD?

Altogether, optimizing management for improved animal robustness against infections, vaccination and mass medication contribute to reduction in the occurrence of BRD. However, despite improvements in our understanding of pathogenesis, the pathogens involved, vaccine technology and means of prevention and treatment, BRD remains one of our most important cattle health concerns in intensive cattle production. The effect of the current preventive measures is not satisfactory, and time is ripe for a novel approach. Can improved biosecurity provide a solution to the problem?

Biosecurity is a set of management and physical measures designed to reduce the risk of introduction, establishment and spread of animal infections or diseases to, from and within an animal population (32). National level biosecurity implies that restrictions on import of live animals and biological products are in place to protect a population from introduction of new infectious agents. External biosecurity refers to measures aiming at preventing introduction of disease into herds. Internal biosecurity relates to limiting transmission of infectious agents between animals or groups within a herd. For BRD, internal biosecurity measures have been reviewed with a focus on factors that limit pathogen exposure within the herds such as vaccination, housing, ventilation and control of other diseases (33).

In the following, herd level biosecurity will refer to external biosecurity at the herd level, which so far has received little, if any, attention regarding BRD. The general herd level biosecurity is relatively low in modern cattle production, also compared to other livestock species such as poultry and swine (34, 35). Few biosecurity measures are usually undertaken, resulting in a constant risk of disease transmission between farms. Implementation of biosecurity measures is hampered by factors such as cost, perceived usefulness, workload and lack of clarity as to how and why measures should be undertaken (34, 36–39). Improved herd level biosecurity can be implemented in single herds, or on a regional or national level. To justify efforts to control BRD through improved herd level biosecurity, the following questions need to be addressed: is BRD a transmissible disease between herds? If so, is it possible to stop the transmissible infectious agents at the farm gate? And can these agents be eliminated from infected herds?

### Is BRD a Transmissible Disease Between Herds?

BRD is a multifactorial disease, and can be caused by a specter of pathogens, often in combination. Viral pathogens such as BRSV, bovine herpesvirus 1, bovine parainfluenza virus 3, bovine viral diarrhea virus and BCoV can cause disease directly, and/or predispose animals to bacterial infections (40–44). Most of these primary BRD pathogens are highly contagious viruses that can easily spread between herds (29, 44), either directly through live animal contact/movement, or indirectly through contaminated environment or fomites brought between herds. The most common bacterial agents are *Mannheimia haemolytica, Pasteurella multocida* and *Histophilus somni* (44–46). These bacteria appear to have lower transmissibility, and bacterial disease in several animals is therefore most likely a result of exposure of animals to the same risk factors, such as virus infection and/or suboptimal environment, at the same time (47). *Mycoplasma bovis* can also contribute to BRD, either as primary or secondary pathogen. Live animal movement seems to be the primary means by which *M. bovis* is transmitted between herds (48).

Variations in the potential for between-herd-transmission between bacterial and viral pathogens affects how effective herd biosecurity is at reducing risk of introduction. The effect of increased biosecurity will therefore vary depending upon which pathogens are present in the area of interest, and their relative contribution to BRD development. The effect of increased biosecurity on the risk of introduction is likely larger for virus than bacterial components of BRD.

Several important BRD pathogens are absent or eradicated in Norway, such as BVDV, bovine herpesvirus 1 and *M. bovis* (49). This highlights the impact of two other viruses in the BRD complex; BRSV and BCoV. Both are highly prevalent in the Norwegian cattle population (31, 50) as they are in most parts of the world, both in intensive and extensive husbandry systems (51, 52). BRSV has been reported responsible for 60% of the BRD epidemics observed in dairy herds (42, 53, 54) and up to 70% in the beef herds (40, 41). In Norway, BRSV has been reported as the main etiological agent causing BRD outbreaks (55). BCoV causes BRD (56) in addition to winter dysentery (contagious acute diarrhea in adult cattle) and diarrhea in calves (52), which further increases the negative consequences of BCoV (57).

Both BRSV and BCoV can be easily transmitted between herds, and epidemics with rapid spread between herds within a region have been reported (57, 58). Modes of transmission are either directly through live animal contact (59) or indirectly via contaminated personnel or utensils brought between herds (60). Herds with limited or no purchase of cattle may also experience outbreaks of BRD, most likely due to introduction of infectious agents by indirect routes, and/or that the causative pathogen was already circulating in the herd (61). Indirect transmission depends upon the stability of the viruses outside the host, which is generally short for enveloped RNA viruses such as BRSV and BCoV. However, there are uncertainties regarding the stability of both viruses. Under laboratory conditions, BCoV remained infective for 2 weeks under cool and moist conditions (62). For both BRSV and BCoV, temporary carriage of virus on fomites has been shown: infective BCoV was detected on fomites (clothes, boots and equipment) 24 h after exposure to virus-shedding calves, while for BRSV, only viral RNA, and no infectious virus, was detected (60). The same study found that personnel in contact with virus-shedding calves carried both BCoV and BRSV RNA on nasal mucosa, but none were positive for infective virions. It was therefore concluded that transmission of virus via human nasal mucosa is likely limited (60). Airborne transmission for BRSV and BCoV has been shown indoors (63) but is most likely restricted to droplet and aerosol spread. Airborne transmission across longer distances, i.e., between farms, has not been described and is likely of limited importance. Transmission of virus from other species to cattle has never been demonstrated and is likely to be of minor importance under normal circumstances.

In conclusion, BRSV and BCoV can be easily transmitted between herds via live animal movements or indirectly via contaminated fomites brought between herds. Airborne transmission and transmission from other animal species such as wildlife, is less likely.

The high impact of BRD in Norway despite freedom from several of the well-recognized pathogens indicates the importance of BRSV and BCoV as key contributors to BRD. Because they are easily transmitted between herds, it can be argued that BRD is a transmissible disease between herds.

### Is It Possible to Stop the Viruses at the Farm Gate?

Because purchase of cattle is an important risk factor for introduction of respiratory pathogens (54, 59), closed herds could be a means to prevent BRD. However, breeding enough replacement animals might not be practical or possible in all systems. Other measures to prevent introduction via live animals to a herd includes purchase of known virus-free animals, routines for safe loading and transportation of animals and isolation of arriving animals. Examples of measures to avoid introduction by people or fomites are establishing infection control sluices including routines for changing boots and clothing upon entering a herd (64), and to avoid bringing contaminated equipment between herds. Safe loading of animals can also prevent indirect transmission.

A recent study from Belgium identified both BRD in general, and especially BRSV infection, as main adult cattle diseases where biosecurity measures should be prioritized (65). Toftaker et al. (59) showed that the odds of being positive for one virus were approximately five times larger if a herd was positive for the other virus, indicating some common risk factors for BRSV and BCoV. Ohlson et al. (64) found a clear association between higher herd biosecurity levels and lower prevalence of herd infection. Implementation of external herd biosecurity routines, such as control sluices, and measures for safe trade are likely to reduce transmission between herds. It would reduce the risk of introduction to the herd where it is implemented, but also the risk of further spread from that herd.

### How Can the Viruses Be Eliminated From Infected Herds?

If introduction of BRSV and BCoV to herds can be avoided, the next question is; what happens with the already infected herds? When BRSV and BCoV cause acute infections in individual animals, the viruses replicate locally in the respiratory epithelial cells and are shed in exhaled air and nasal secretions (43, 51). BCoV also infects enterocytes and is excreted in feces (43). Experimental studies have shown shedding of BRSV from day three to nine post infection (46), and from day two to ten for BCoV (66, 67). Viral RNA can be detected for an extended period (67, 68), but might not represent infective virus. Both infections give short-lived immunity (69–71). Introduction of virus to a herd usually results in rapid spread and high within-herd prevalence. This is particularly seen during the winter season (50, 72). Depending on factors such as herd size, management and the immunity of the herd, viruses may continue to circulate due to subclinical infections in naïve animals and/or reinfections with viral shedding in seropositive animals (43, 73).

Some data indicate that persistence of BRSV and BCoV in individual animals is possible. Infective BRSV has been isolated from lymph nodes 71 days post infection (74). BCoV persistence has been demonstrated in cell culture (75). Long-term PCR positivity in calves has been shown in one experimental study, but transmission potential was not confirmed by virus isolation/sentinel trials nor was sequencing of virus done to exclude new infection (76). The epidemiological role of such persistence in individual animals is somewhat unclear, but transmission of reactivated virus to susceptible animals has never been shown.

In a longitudinal study, repeated sampling of dairy herds showed that 32–42% of the herds changed their BRSV antibody status from positive to negative based on pooled calf sera during a 6-month time period (50). Similar results have been found for both BRSV and BCoV in Swedish dairy herds (77). This indicates rapid self-clearance of virus from herds without specific interventions. Molecular epidemiology supports this view—virus varies both temporally and spatially between outbreaks, suggesting that outbreaks are caused by introduction of new virus rather than through reactivation or the existence of carrier animals (78–80). This implies that with the current herd size and management conditions in the Nordic countries, herds can self-clear from virus if new introduction is avoided.

## Possible Alternative Approaches to Combat BRD at a Regional, or National Level

Given the substantial impact of BRD and unsatisfactory results of existing preventive measures, alternative strategies to combat BRD are urgently needed. Stakeholder interest is of fundamental importance to succeed in animal disease control. Furthermore, a herd level control program requires a cost efficient and reliable method for classification of herds as well as adequate disease monitoring.

### Producer Attitudes to Regional Disease Control Efforts

A systematic approach to BRD control implies systematic measures to reduce the incidence of the transmissible infectious agents, and in the Norwegian example, for BRSV and BCoV infection on herd level, implemented on a sectoral, regional or national level. Is this achievable? The answer depends on cultural and structural conditions of the cattle industry in the area of interest. Introduction of virus could, in principle, be prevented in any herd. However, a synergetic effect can be acquired if measures are implemented by most of the herds in an area or country. How successful a herd's biosecurity measures are also depend upon the infectious pressure from the outside. If this is reduced due to better biosecurity in surrounding herds, the benefit will be mutual. A central, joint organization to run a program through is therefore an advantage. In Norway, the largest dairy company (TINE SA) is a co-operative owned by the producers and 96% of the dairy herds report to the Norwegian dairy herd recording system, where membership is voluntary (81). This probably contributes to high compliance in voluntary control efforts established by the industry.

A producer's willingness to implement management strategies or disease control programs has been found to be influenced by individual values and beliefs, by other producers, the industry or the government (82). Earlier positive experiences with disease-control makes it easier to introduce new projects. For example, the Norwegian producers likely have a stronger willingness to participate in joint disease-control efforts, also for non-reportable diseases, due to the successful elimination of bovine virus diarrhea from the cattle population in 2006 (83). This program was established in 1992 by the dairy- and breeding organizations, in collaboration with the animal health authorities. Ringworm due to *Trichophyton verrucosum* has nearly been eliminated due to an eradication program that combined vaccination and zoosanitary measures (84). Cost-benefit analyses of previous national control programs in the dairy cow and goat sectors have proven that the efforts paid off (85, 86). Motivation is crucial and necessary in order to succeed in implementing measures that requires extra effort.

### Herd Classification and Disease Monitoring

In order to monitor the disease situation at the population level, a suitable classification system for herds is needed. Different sample material can be used, and a diagnosis can be made on individual animal level or at the group/herd level. Generally, infection with BRSV and BCoV can be diagnosed by detection of virus, viral antigen, or viral RNA in tissues, secretions, or excretions of infected animals (43, 78, 87). Antiviral antibodies are usually detected by commercial ELISA tests and there is a good agreement between titers in serum and milk for both viruses (88).

During viral shedding, nasal swabs (BRSV/BCoV) and feces (BCoV) can be used for antigen detection or for genome detection by RT-PCR (87). Antibody detection can also be used during outbreaks but requires paired acute and convalescent samples. Serological investigations are used for retrospective diagnostics and screening studies (prevalence), often at the herd level. Because animals are seropositive for many years after infection (66, 89), seropositivity is a slow-changing indicator which indicates previous exposure to virus, but not necessarily presence of virus.

The herd-sensitivity and herd-specificity of a diagnostic test is influenced by the basic performance of the test, the within-herd prevalence and the number of animals tested. Misclassification can arise as a result of imperfect test performance or changes in status after testing. Imperfect test performance could also be due to a suboptimal test-regime with regard to which, and how many, animals are tested.

The interpretation of testing will depend on the age and number of animals sampled. Bulk tank milk serology can provide an estimate of herd-level seroprevalence of BRSV and BCoV (90). The method is cheap, but the result only reflect that there has been virus present in the herd during the last years. Sampling of a group of younger animals has also been used, with the assumption that the selected animals are representative for their age group in the herd e.g., pooled milk samples from primiparous cows. As they usually are 2–3 years old, the sample will reflect a herd's infection-history 2–3 years prior (77). Serum from a group of calves under 1 year of age will indicate virus circulation within the last year, if calves young enough to have maternal antibodies are excluded. Classification of herds based on serological analysis of a group of animals is therefore possible, and the different options have pros and cons with regard to cost and value. We reiterate that the gap between seropositivity and virus presence is considerable. A seronegative herd is, on the other hand, a good indicator of a virus-free herd, and in the context of a control program, finding the free herds might be most important.

## The Norwegian BRSV and BCoV Control Program

The recently launched Norwegian BRSV and BCoV control program is presented as an example of a national level control program based on systematic improvement of external biosecurity at the herd level. The program contains no vaccination or mass-treatment. A brief description of cattle production in Norway is included for context, followed by an outline of the chosen method for herd classification and the applied biosecurity measures.

Milk production in Norway is extensive and based on small, mostly family run-farms. The number of dairy herds is around 8,300, with an average herd size of 27 cows in 2018. For members of the Norwegian dairy herd recording system, production data is available to advisors and veterinarians. Many producers rear their own heifers and keep bull-calves for slaughter, which means that young stock and adult cows are often kept in the same or nearby facilities. The number of beef herds is 3,600. These are predominantly suckler-cow herds with an average number of 23 cows, which rear their calves until slaughter (16). There is no tradition for specialized beef production, but over the last decade, several cow-calf operations with beef-breeds (or beef-crosses) and a few fattening units have been established.

In a nationwide study of 134 randomly selected Norwegian dairy herds, Gulliksen et al. (31) found 31.2% of the calves in 71.1% of the herds to be positive for antibodies to BRSV, while the same numbers for BCoV were 39.3% and 80.7%, respectively (31). Toftaker et al. (59) found the prevalence of seropositive herds in bulk tank milk to be 46.2% for BRSV and 72.2% for BCoV in two counties in the western part of Norway. Large variations were found in prevalence across the study region, with high risk clusters as well as overall geographic trends. Negative herds were found in close proximity to positive herds (59). About 40% of the herds were positive for antibodies to both viruses, while 22% were negative for both.

The control program for BRSV and BCoV was initiated by a joint cattle industry and launched in Norway in 2016. The goal of the program is to reduce the occurrence of BRSV and BCoV in the national cattle herd. A key feature is to classify all herds according to BRSV/BCoV antibody status (sero-positive or -negative) and protect animals in both positive and negative herds from infection through herd biosecurity measures. Vaccination or antimicrobial treatment is not included in the implementation plan, and vaccination against BRD is usually not practiced. Knowledge of herd status is assumed to motivate producers to implement the recommended measures for prevention of virus introduction. Furthermore, sero-negative herds who can document specific additional biosecurity measures are eligible for financial incentives.

The costs of the Norwegian control program are shared between producers and the industry. The dairy industry financed the initial screening of dairy herds and the meat industry financed testing of beef herds. After that, the cost of one testing per year per herd is covered by the industry. A project leader is employed by the industry partners and responsible for information flow and education of veterinarians, producers and others within and outside the organizations.

### Classification of Herds

**Figure 1:**
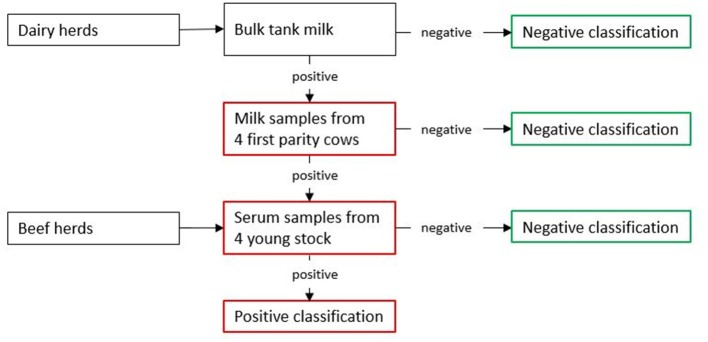
Classification of herds in the Norwegian control program for bovine respiratory syncytial virus (BRSV) and bovine coronavirus (BCoV). Samples of milk and serum are analyzed for antibodies against BRSV and BCoV using a BCoV/BRSV multiplex immunoassay.

The principle of herd classification in the control program is illustrated in Figure 1. Specifically, dairy herd classification is based on serological examination of (1) bulk tank milk, (2) pooled milk samples from first-parity cows, or 3) pooled serum from young stock. If testing of bulk tank milk indicates sero-positivity, producers are encouraged to test pooled milk samples from four first-parity cows. If this yields a positive result, testing of pooled serum from young stock is recommended. Only homebred, unvaccinated animals above 180 days of age (to avoid maternal antibodies) are tested in (2) and (3). If four animals are not available, three and two may be used. Beef herds are tested using young stock only. The system is the same regardless of housing conditions or size.

All samples are tested with a new multiplex immunoassay for BRSV and BCoV antibodies (MDV-Enferplex BCV/BRSV multiplex from Enfer Scientific, Naas, Ireland). The sensitivity and specificity for the bulk tank application of the test have been estimated to 94.4 and 90.6 for BRSV and 99.9 and 93.7 for BCoV, respectively (90).

All producers automatically receive free material for collection of samples. Untested herds and herds with inconclusive results are classified as positive in the program. A negative status is valid for 1 year, and the producers are automatically reminded when new testing is recommended. Purchase of animals from positive herds automatically leads to positive status.

### Biosecurity Measures

The recommended biosecurity measures aim at protecting herds from introduction of virus via direct (live animals) and indirect (people and fomites) routes. All producers are encouraged to avoid live animal contact between negative and positive herds by purchasing animals or sharing pastures with animals only from sero-negative herds. Live-animal trade is organized by the producer organizations, both for replacement animals and for animals shipped to slaughter. Since the launch of the control-program, separate transport vehicles have been used for animals from negative and positive herds. Farmers are encouraged to build suitable loading areas for shipment of live animals. Furthermore, improved external biosecurity is encouraged by implementing restricted human access into herds. There is a legal requirement to provide sluices where veterinarians, AI technicians, advisors, claw trimmers, service people and others can change to protective clothing and footwear provided by each herd. Advisory support from the program is provided to ensure a feasible design of these sluices. In general, the advice is for the herd to provide clothing and footwear for visitors, washing facilities with cold and hot water and suitable storage areas for equipment.

To encourage compliance with the control program, herds can acquire a “Healthy herd status” by fulfilling a set of specific criteria. These criteria include having a sero-negative status for both BRSV and BCoV. In addition, the herd needs a veterinary certificate confirming high external biosecurity through the implementation of a physical barrier sluice. A loading-area for shipment of live animals to and from the herd is also required, to enable the truck driver to access the animals without entering the barn. A “Healthy herd” status is rewarded with an increase of approximately 10% in price when selling young-stock and breeding animals.

A final measure to be mentioned is the establishment of a “hot-line,” where producers report episodes of diarrhea or respiratory disease by phone. This is done to enable rapid discovery of possible outbreaks, and a notification leads to warning of relevant personnel (e.g., field practitioners and milk truck drivers) such that necessary precautions can be taken to avoid further disease transmission and increase the vigilance in the area.

## Discussion

We have presented arguments for biosecurity-based control of BRD and outlined the ongoing Norwegian control program for BRSV and BCoV. We argue that successful population-level disease control is possible through external herd level biosecurity measures but that several conditions must be met.

Generally, the requirements for initiating a control program will differ according to biological factors (species affected, zoonotic potential, reservoir, population structure and basic characteristics of the infectious agents etc.), possible control measures (movement control, stamping–out, isolation, vaccination etc.), availability of technical tools (diagnostics tests, treatment) and socioeconomic considerations (91). Lindberg and Houe (92) concluded that for successful control of bovine viral diarrhea virus (BVDV), three elements are necessary: basic biosecurity, elimination of virus from infected herds and monitoring to evaluate progress and detect new infections/reinfections. Despite considerable biological differences between BVDV and BRSV/BCoV, the same three same elements are also fundamental in the control of BRSV and BCoV.

The first element, biosecurity, is the primary focus in the control program. The aim is to reduce risk of introduction of virus both through live animals, people and fomites. A critical point is the efficacy of the recommended protective measures. This may differ according to management system and herd structure. For example, large herds have been shown to have more visitors and thereby more indirect contacts compared to smaller herds (34). This can partly explain why large herd size is a frequently reported risk factor for herd level positivity to both BRSV and BCoV (58, 59, 64, 93, 94).

The effect of the recommended protective measures also depends upon the compliance to these, where the motivation among stakeholders, veterinarians and producers is crucial. It is also an ongoing measure that needs to be nourished over time. Basic education, as well as a continuous flow of updated information, is necessary. Knowledge about the occurrence of the infections is useful to motivate action. The impact of BRD is well-recognized among farmers and veterinarians in Norway, and they are usually aware that BRSV and BCoV are the primary pathogens, and that BCoV also causes winter dysentery. This probably makes it easier to motivate the producers for control of BRSV and BCoV in Norway compared to countries with other Specters of BRD pathogens. For BRSV and BCoV, the documented varying prevalence, and presence of negative herds in high-prevalence areas (50, 59), shows the Norwegian producers that it is possible to stay negative also if neighbors are positive. For regions with higher prevalence of BRSV and BCoV, an important step forward would be to perform an antibody-screening with a classification method that gives a recent picture, for example investigation of first-parity cows or young stock before concluding that all herds are positive. For countries with severe problems due to other BRD pathogens such as BVDV, *M. bovis* and IBR, it is probably wise to focus on these pathogens first. However, the preventive measures will generally have positive effect on the transmission of many other infections.

The second element, elimination of virus from infected herds, receives little attention in the program as self-clearance is regarded likely. This is probably more effective in small herds, and the small average herd size in Norway is therefore an advantage. In larger herds, naïve cattle in sufficient numbers might be available all the time, and both acute, subclinical infections and possible persistent infections are more likely. Altogether, control might be more challenging in areas where herds are larger, and more intense monitoring might be necessary. Nevertheless, biosecurity-based control might still succeed if new introduction of virus is avoided, as it will most likely be a question of time before virus cease to circulate also in larger herds.

The third element, monitoring of progress, is based on the feasibility of the classification of herds, and the frequency of the testing. There is a need for herd-level diagnostic tools that accurately classify the herds in a cost-efficient manner. Serological investigations will result in an overestimation of prevalence, as earlier discussed. In the Norwegian test-regime the small average herd size might cause few first-parity cows or calves to be available, consequently reducing the herd-sensitivity. The within-herd prevalence is to some degree unknown and probably variable between herds, and within groups in the herds, which further complicates the matter. In the control program, the test-result is valid for a full year. The probability of virus introduction after classification is considerable, particularly in herds that purchase animals. An updated herd-classification based on the combination of bulk milk tank testing, herd size, information on animal movements and geographical location has been shown to provide a more accurate estimate of herd status (95) and could potentially improve progress of the program.

Altogether, herd size influences all the three fundamental elements discussed here. It is also where the Norwegian situation differs considerably from most European countries. Our average dairy herd comprises 27 cows and suckler-cow herds 23, and there is an absence of feed-lots as well as few and small fattening herds. In addition, herd size might also influence the time until a new infection is detected. In Norway, the number of animals tested is the same regardless of size. In herds with many animals a control program with a more intense diagnostic test regime regarding both number of animals tested and frequency of testing, might be necessary. Herd sizes are increasing in Norway, which coincides with an increase in the recorded number of infectious diseases (96). Infection control in areas with larger herds is therefore likely to be more challenging, but even more necessary and rewarding if successful.

Stakeholders and producers are obviously concerned with the costs related to a control program; is it worth it? The financial losses due to BRSV and BCoV in Norway were analyzed by the industry prior to onset of the program. This included the available knowledge of the viruses' effect on BRD and winter dysentery, and the costs of running a control program were weighted against the impact (not published). It was concluded that controlling BRSV and BCoV would be cost-efficient and should be prioritized. There are several uncertainties in such an analysis. In a study from France, the authors assumed that a reduction of BRD incidence between 20 and 50% was a realistic outcome to expect from improvements in farm biosecurity (13), but further studies that link epidemiology and livestock productivity in a larger scale is needed.

The situation in Norway with few transmissible and notifiable diseases highlight the large impact of BRSV and BCoV. Control of these highly contagious viruses require a systematic approach, and a cooperative culture with a common goal. Previous experience from systematic eradication and control of other diseases might have contributed to a culture for disease control through prevention and joint efforts in Norway. Successful control of BRSV and BCoV here could motivate to action also in other countries. Effects on public health is a profound reason for animal disease control. The expected benefits is considerable regarding the usage of antimicrobials and antimicrobial resistance, in agreement with the present OIE strategy (90). Another expected “by-product” of the control program is the likely reduction of infections caused by other pathogens transmitted via the same routes, both endemic and emerging pathogens. The Norwegian BRSV and BCoV control program indicates a way forward in how to achieve improved animal health and welfare.

## Concluding Remarks

Antimicrobial resistance is a major public health threat. Growing concerns regarding the environmental impacts of livestock calls for new and innovative measures to prevent endemic diseases, and thereby improve the sustainability of the cattle industry. An alternative strategy to combat BRD is urgently needed. We believe it is both desirable and possible to control BRSV and BCoV, and subsequently reduce BRD, through biosecurity measures. The Norwegian initiative represents a new way of thinking that will likely have wider implications. The ultimate goal is improved animal health, welfare and a reduction in antimicrobial usage in the cattle sector as well as a more effective production.

## Author Contributions

Development of concept was done during a walk-shop where all authors contributed ideas and input. Drafting of the manuscript was led by MS, with contributions and critical review from all authors. All authors have read and approved the final product.

### Conflict of Interest

The authors declare that the research was conducted in the absence of any commercial or financial relationships that could be construed as a potential conflict of interest.
